# Implementation of national guidance for self-harm among general practice nurses: a qualitative exploration using the capabilities, opportunities, and motivations model of behaviour change (COM-B) and the theoretical domains framework

**DOI:** 10.1186/s12912-023-01360-3

**Published:** 2023-12-01

**Authors:** Jessica Z. Leather, Chris Keyworth, Nav Kapur, Stephen M. Campbell, Christopher J. Armitage

**Affiliations:** 1 NIHR Greater Manchester Patient Safety Translational Research Centre, The University of Manchester, Manchester Academic Health Science Centre, Jean McFarlane Building, Oxford Road, Manchester, M13 9PY UK; 2 Manchester Centre for Health Psychology, Division of Psychology and Mental Health, School of Health Sciences, University of Manchester, Jean McFarlane Building, Oxford Road, Manchester, M13 9PY UK; 3 The School of Psychology, The University of Leeds, Woodhouse Lane, Leeds, UK; 4 Centre for Mental Health and Safety, University of Manchester, Manchester Academic Health Science Centre, Manchester, UK; 5 Greater Manchester Mental Health NHS Foundation Trust, Manchester Academic Health Science Centre, Manchester, UK; 6 Centre for Primary Care and Health Services Research, School of Health Sciences, University of Manchester, Manchester, UK; 7 Manchester University NHS Foundation Trust, Manchester Academic Health Science Centre, Manchester, UK; 8 NIHR Manchester Biomedical Research Centre, Manchester University NHS Foundation Trust, Manchester Academic Health Science Centre, The Nowgen Centre, Manchester, UK; 9 Department of Public Health Pharmacy and Management, School of Pharmacy, Sefako Makgatho Health Sciences University, Molotlegi Street, Pretoria, 0208 South Africa

**Keywords:** Self-harm, General practice, Practice nurse, Primary care, Evidence-based guidelines, Tailoring

## Abstract

**Background:**

Patients who self-harm may consult with primary care nurses, who have a safeguarding responsibility to recognise and respond to self-harm. However, the responses of nursing staff to self-harm are poorly understood, and opportunities to identify self-harm and signpost towards treatment may be missed. It is unclear how to support nursing staff to implement national guidelines.

**Aims:**

Among primary care nursing staff to: [1] Examine reported barriers and enablers to nurses’ use of, and adherence to, national guidance for self-harm; and [2] Recommend potential intervention strategies to improve implementation of the NICE guidelines.

**Methods:**

Twelve telephone interviews partly structured around the capabilities, opportunities and motivations model of behaviour change (COM-B) were conducted with primary care nurses in the United Kingdom. The Theoretical Domains Framework was used as an analytical framework, while the Behaviour Change Wheel was used to identify exemplar behaviour change techniques and intervention functions.

**Results:**

Nursing staff identified a need to learn more about risk factors (knowledge), and strategies to initiate sensitive conversations about self-harm (cognitive and interpersonal skills) to support their professional competencies (professional role and identity). Prompts may support recall of the guidance and support a patient centred approach to self-harm within practices (memory, attention, and decision making). GPs, and other practice nurses offer guidance and support (social influences), which helps nurses to navigate referrals and restricted appointment lengths (environmental context and influences).

**Conclusions:**

Two converging sets of themes relating to information delivery and resource availability need to be targeted. Nine groups of behaviour change techniques, and five intervention functions offer candidate solutions for future intervention design. Key targets for change include practical training to redress conversational skill gaps about self-harm, the integration of national guidance with local resources and practice-level protocols to support decision-making, and creating opportunities for team-based mentoring.

**Supplementary Information:**

The online version contains supplementary material available at 10.1186/s12912-023-01360-3.

## Background

General practices represent a first point of contact for many people who self-harm; most often to seek ongoing care for self-harm as a consequence of mental health difficulties, but patients also present to primary care immediately following an episode of self-harm [1, 2]. Rates of self-harm recorded in primary care have risen over the past decade, particularly in young people and older adults [3, 4]. Despite being a robust risk factor for subsequent death by suicide [5, 6], self-harm remains an overlooked and often misunderstood behaviour outside of specialist mental healthcare settings [7, 8]. To address this, a key component of the United Kingdom (UK) government’s suicide prevention strategy has been to call for appropriate suicide and self-harm training for all staff in primary care settings, including General Practitioners (GPs) [9, 10]. The role of GPs in identifying and managing patients has been a priority for self-harm research in primary care [2, 11, 12], but the contributions of other primary care staff has received less focus.

Although the makeup of general practice teams in the UK can vary depending on size and location, patient-facing roles typically include partner, salaried, and trainee GPs, practice nurses, advance nurse practitioners, and healthcare assistants. Some teams also include onsite pharmacists and dispensers. While pharmacy staff have been recognised as important gatekeepers to prevent self-poisonings [13] and other primary care providers such as paramedics frequently encounter patients who self-harm [14, 15], general practice is an important setting for intervention because many people who self-harm in the community do not present to emergency services [16, 17]. As a result, GPs and practice nurses are often a first point of contact for people who self-harm in the community [1, 3, 4, 18]. However, potentially hazardous non-adherence to national self-harm guidance has been documented in general practice [19, 20], and self-reported implementation of these guidelines is poorer among staff who are not GPs [21]. This indicates that nursing staff in general practices may benefit from support to follow national guidance for self-harm.

Scant information is available about what typically happens when a patient attends general practice to seek care relating to self-harm, or how frequently such occurrences take place with a nurse. A study of young people’s medical records found that patients tend to seek to see different healthcare professionals depending on the nature of their self-harm; patients that self-injure prefer to consult with a practice nurse, while people that have self-poisoned make appointments directly with their GP [18]. Nursing staff are practice team members with a duty of care towards patients, professional registration requirements for the use of evidence-based care, and considerable autonomy [22]. These roles provide potential opportunities to identify and signpost patients who may be at risk of further self-harm, in addition to providing ongoing care for patients whose self-harm has already been recorded. Therefore, it is paramount that the experiences of these staff members are understood, to ensure best care for patients and that their practice is congruent with the expectations of national guidance and nursing competencies.

The National Institute for Health and Care Excellence’s (NICE) guidance for self-harm has recently been updated into a single guideline [22]. The previous guidance is largely consistent with the combined guidance, stating that all clinical and non-clinical employees working for a primary care service should be provided with appropriate training to understand and care for people that have self-harmed [23–25]. The most recent update to the Nursing and Midwifery Council (NMC) standards of proficiency for registered nurses includes competencies related to suicide prevention [26]; specifically the knowledge to recognise and assess self-harm risk, and the skills to initiate interventions for people who self-harm. In addition to providing direct care, nursing staff may encounter patients that present following an episode of self-harm, who need to be escalated for an initial assessment by a GP [27]. However, healthcare professionals who qualified before the new curricula were published in 2018 may not be aware or have achieved these competencies, and there are concerns that existing training programmes do not adhere to national self-harm and suicide prevention competency frameworks [28, 29]. Such gaps in education create barriers to implementing national self-harm guidelines, since staff who are poorly informed about self-harm are less likely to identify patients who are at risk [30, 31].

Beyond skills and knowledge, a lack of training about self-harm negatively impacts nurses’ confidence to address self-harm routinely, due to fears that confronting sensitive mental health topics incorrectly would offend or distress their patients [18, 31]. A recent nationally representative survey of healthcare professionals found that under one third of respondents (30.6%, *n* = 312) had taken part in any training about self-harm [21]. Practice nurses followed the guidance with 33% of patients perceived to be at risk of further self-harm, compared to 61% of GPs. A previous survey found that asking about self-harm was not routine practice in primary care, suggesting that opportunities to intervene may be missed by healthcare professionals because of an absence of habits [32]. Environmental barriers to guideline-adherent practice in primary care include a high volume of patients, short appointment times, and difficulties making referrals to secondary mental health services [2, 33, 34]. Since these findings are chiefly derived from GP samples, there remains a need to establish what unique barriers nursing staff face within this healthcare setting, using comprehensive theoretically-grounded frameworks. To change healthcare professionals’ behaviour, implementation strategies require a foundational understanding of the multifaceted determinants of guideline-congruent practice, as informed by behaviour change theory, to identify drivers and appropriate strategies for change [35, 36].

The capabilities (C), opportunities (O), and motivations (M) model of behaviour change (B; COM-B) [37] provides an accessible approach to conceptualise the environmental, social, affective, and cognitive influences on behaviour. The COM-B stipulates that behavioural drivers can be categorised into its six components, which were distilled from numerous theories of behaviour change [38]. However, the breadth of these components limits its utility for intervention design because it does not provide an adequate level of detail about the determinants of behaviour. An extension of this model known as the Theoretical Domains Framework (TDF) [39] offers a solution. The TDF was purposely designed for implementation research by elaborating on the components of the COM-B model with a framework comprising 14 domains [36, 40]. The TDF is part of the Behaviour Change Wheel for intervention development which has the COM-B model at its centre [38]; as a result, the TDF offers a systematic approach to identify corresponding intervention functions, behaviour change techniques, and policy categories to develop theory-based behaviour change interventions [41]. This approach has been used as a basis for intervention development for practice nurses in relation to cervical screening [42] and consultations for osteoarthritis [43].

While primary care nurses encounter patients that self-harm in primary care, few implement national guidance for self-harm and opportunities to identify and signpost towards care for patients at risk of further self-harm may be missed. The present study aimed to: [1] examine the barriers and enablers to primary care nursing staff’s use of, and adherence to, national guidance for self-harm, and [2] recommend potential intervention strategies to improve implementation of the NICE guidelines and nursing competencies relating to self-harm by primary care nurses.

## Methods

### Design

Semi-structured telephone interviews were conducted with primary care nurses based in general practice. A topic guide structured around components of the COM-B model [37] was produced, which was derived from an existing schedule created for use with healthcare professionals [44] (Additional file 1). Structuring interview questions around the COM-B provided: (a) theoretically-grounded questions to explore drivers of guideline implementation, (b) the option to use the TDF as an analytical framework to interpret themes in the data, and (c) the ability to connect components of the COM-B model to the TDF framework to precisely identify barriers and enablers to implementing national guidelines for self-harm. Using open-ended questions structured around COM-B components instead of TDF domains enabled respondents to discuss drivers of their own behaviour spontaneously; this is advantageous because it provides opportunities for participants to describe barriers or enablers that may not be sufficiently captured by the TDF [45].

### Participants

A purposive sample of 12 GP practice nurses were recruited to this study. Participants had already taken part in a cross-sectional survey (administered by a survey panel company) examining implementation of the NICE guidelines for self-harm [21], and had expressed an interest in taking part in follow-up research. All participants had qualified prior to the addition of the of suicide prevention competencies to the NMC’s nursing proficiencies [26]. The final interviews generated no new data, suggesting data saturation had been achieved.

### Data collection

Ethical approval was granted in February 2019 by a university ethics committee (Ref: 2019–5456-9504), and data collection took place between April 2019 and May 2019. Two members of the survey panel company (one male, one female) conducted the interviews; external interviewers were utilised to minimise potential response biases arising from the interviewees’ professional relationships [46]. Both interviewers were provided with an interview topic guide and summary information sheet about the NICE guidelines for self-harm for reference (Additional file 2). The information sheet advised the interviewers to (a) use open-ended questions to allow drivers of guideline implementation to be explored spontaneously; (b) be cautious when asking about current practice to mitigate social desirability or professional identity biases, and (c) ask participants to provide examples of any instances where they encountered patients who had self-harmed [38]. Invitation emails were distributed following completion of the online survey [21]; the survey panel company incentivised potential participants with a points-based reward system [47]. The telephone interviews were recorded and transcribed verbatim by the company, then anonymised and delivered to the research team for analysis. Informed consent was obtained before each interview. No personally identifiable participant data was shared with the research team, to adhere to YouGov’s General Data Protection Regulation (GDPR) regulations.

### Analysis

A framework approach [48] was adopted to map the data onto appropriate TDF domains using Microsoft Excel. Interview scripts were analysed by two members of the research team (JZL and CK), who are health psychology researchers. The researchers reviewed each other’s analysis during ongoing meetings. Unanimous agreement was reached about the codes allocated to the data, suggesting the coding process was consistent between both researchers. To ensure the TDF was an appropriate analytical framework, CK reviewed data from the first 25% of interviews matched to domains by JZL. The researchers agreed about the majority (> 60%; [39]) of domains assigned to the data, and areas of disagreement were resolved through discussion to reach a consensus about appropriate domains.

Deductive (first level) coding was used to identify, record, and categorise occurrences of TDF domains in the data (i.e.: directed content analysis; [49, 50]). Likewise, data relating to COM-B components were coded and assigned to their corresponding TDF domains (as described in [38]). Key domains were selected based on: [1] their prominence in the data (mentioned by > 60% of participants) and [2] whether their significance was described spontaneously by participants. These two criteria have been used in previous research to identify salient domains (e.g.: [44, 51]). Inductive (second level) coding was then undertaken to generate explanatory themes for the key domains [39].

Finally, the Behaviour Change Wheel [38] was utilised to interpret the domains and identify functions and behaviour change techniques to illustrate how the findings could be used to inform intervention design [41] by JZL and CJA, who are experts in health psychology. The Behaviour Change Wheel is an amalgamation of nineteen frameworks of behaviour change interventions, and uses the COM-B as its central hub. It contains nine categories of intervention functions to address deficiencies in capabilities, opportunities or motivations (e.g.: Incentivisation), and seven policy categories that could enable those interventions (e.g.: Fiscal measures, [38]). Exemplar behaviour change techniques (BCTs) and intervention functions recommended by the Behaviour Change Wheel have been operationalised by providing candidate intervention strategies to illustrate how the techniques could be used to improve implementation of the NICE guidelines for self-harm.

## Results

**Fig. 1:**
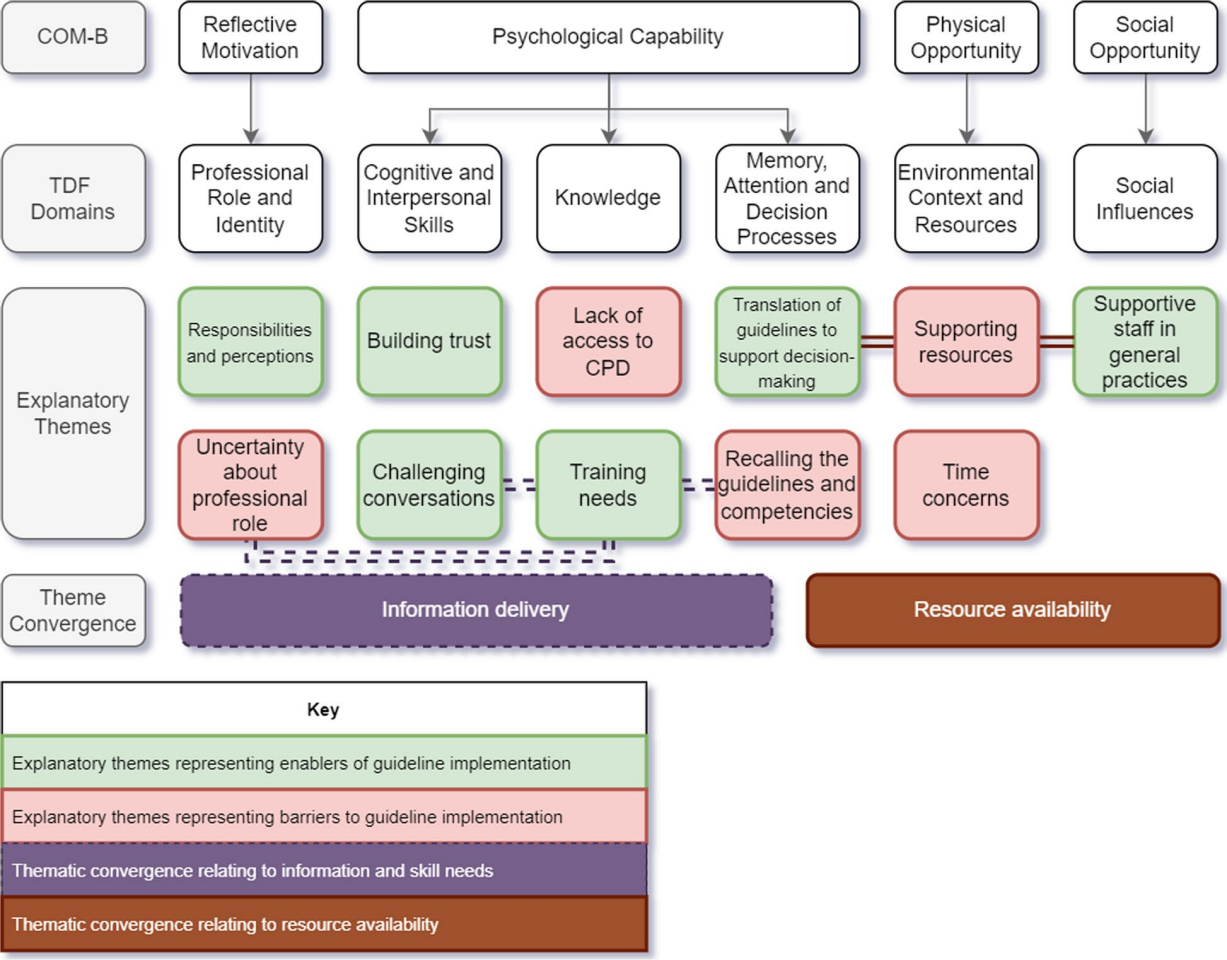
Barriers and enablers to implementing the NICE guidelines for self-harm

Participant demographics are detailed in Table 1. The sample (*n* = 12) comprised primary care nurses working in GP surgeries. Length of interviews ranged from 18–43 min (mean length 33 min). Results are presented by theoretical domains and explanatory themes; a summary is presented in Table 2, while key findings are illustrated in Fig. 1.

**Table 1:** Participant demographics (*n* = 12)

**Variables**	N	%
**Gender**		
Female	12	100.00
**Age**		
35–44	3	25.00
45–54	2	16.67
55–64	6	50.00
Did not state	1	8.33
**Ethnicity**		
White British	9	75.00
Chinese	1	8.33
Other ethnic group	1	8.33
Did not state	1	8.33
**Years in profession**		
First year of practice	1	8.33
7–10 years	2	16.67
16–20 years	1	8.33
Over 20 years	8	66.67
**Work Setting**		
GP practice	12	100.00
**Professional Role**		
General Practice Nurse	10	83.33
Lead General Practice Nurse	1	8.33
General Nurse Practitioner	1	8.33

**Table 2:** Summary of key findings

COM domain	TDF domain	Description of domain	Example quotes	Intervention function	Individual BCTs	Candidate interventions
Psychological Capability	Knowledge	Knowledge gaps were identified pertaining to self-harm risk factors and protocols. Nurses did not perceive a need for in-depth learning about self-harm, instead wanting to improve their awareness of the self-harm guidelines	“They touch upon things like mental health and our role in searching out signs and things like that but it would, kind of, half a lecture. I don’t think it’s quite focussed on enough.” (Practice Nurse 1)	• Education	• Prompts/cues • Information about health consequences • Information about emotional consequences • Information about antecedents	Education about the positive impacts on health associated with arranging a psychosocial assessment following self-harm (IF: Education; BCT 5.1: Information about health consequences)
	Cognitive and Interpersonal Skills	Nurses were highly skilled at building trust with patients. Practical advice was needed about starting conversations about self-harm in ways that preserve patient trust and rapport	“I think it’s harder for nurses because we don’t always have the skills because we have more of a broad ranging set of skills.” (Practice Nurse 10)	• Training	• Demonstration of the behaviour • Instruction on how to perform a behaviour • Behavioural rehearsal/practice • Feedback on the behaviour	Training nurses to be able to start conversations about self-harm during appointments when it is safe and appropriate to do so (IF: Training; BCT 4.1: Instruction on how to perform the behaviour)
	Memory, Attention, and Decision Process	Translation of the guideline content into practice- or Trust-level protocols would support decision-making. Brief reminders, akin to annual training packages, would support recall of the guidance	“There’s so much think about in general practice, and we can’t have the answers to everything, you know, so sometimes it’s good to have pathways or guidance or standards or somewhere where you can look to find some guidance really.” (Practice Nurse 5)	• Training • Environmental restructuring • Enablement	• Habit formation • Prompts/cues • Adding objects to the environment • Restructuring the physical environment	Develop an in-practice protocol for staff to follow when they encounter a patient who has self-harmed or is at risk of self-harm (IF: Environmental restructuring; BCT 12.1: Restructuring the physical environment)
Physical Opportunity	Environmental Context and Resources	Short appointments hampered opportunities to adequately discuss self-harm. Barriers to organising referrals consistent with the guidelines included extensive written communications with secondary mental health services, and unclear waiting times	“That’s not a ten-minute appointment, so it would take longer. It would put pressure on your other colleagues, who would maybe have to pick up on your other patients, it would make me feel bad because I know that I’ve got patients waiting, but I need to obviously concentrate on the job in hand.” (Practice Nurse 7)	• Environmental restructuring • Enablement	• Restructuring the physical environment • Restructuring the social environment • Action planning	Simplify the paperwork required to organise a mental health referral to reduce the time burden of written referrals. (IF: Environmental restructuring; BCT 12.1: Restructuring the physical environment)
Social Opportunity	Social Influences	Support from colleagues in general practices enabled participants to implement the guidelines. ‘On-call’ systems and designated mental health staff were sought out during encounters with patients at risk of self-harm. All-staff meetings are opportunities to disseminate updated guidance	“The biggest help is having another person who I can get to come and have a conversation as well. So, being able to whip across the corridor and say to the doctor or the other practice nurse: ‘Will you just come and have a word with this patient as well and see what you think’.” (Practice Nurse 4)	• Environmental restructuring • Modelling • Enablement	• Restructuring the social environment • Demonstration of the behaviour • Social support (practical)	Designating a member of staff as the lead for mental health or safeguarding, to be contacted if a patient presents with self-harm or is believed to be at risk of self-harm. (IF: Enablement; BCT 12.2: Restructuring the social environment)
Reflective motivation	Professional/ Social Role and Identity	Nurses and healthcare assistants are perceived to be approachable, which creates opportunities to identify and signpost patients who self-harm. While a sense of duty towards patients motivated participants to implement guidelines, some argued that their role restricted the actions they could take beyond signposting	“I think we need to keep an eye out, sort of, for any evidence or any concerns that we have and I think it’s our responsibility to either, if we feel appropriately trained to do so, or if we feel it appropriate for us to do so, to raise that issue with the patient.” (Practice Nurse 1)	• Education • Modelling	• Information about antecedents • Information about others’ approval • Demonstration of the behaviour	Provide information to nurses about how patients want to talk about self-harm; specifically what patients do and do not find helpful. (IF: Education BCT 6.2: Information about others’ approval)

Note: No BCTs are associated with ‘memory, attention, and decision processes’ and ‘professional role and identity’, so BCTs were selected from the relevant intervention functions

*IF* Intervention Function, *BCT* Behaviour change technique

Convergence between explanatory themes is depicted in Fig. 1 by connecting lines; two groups of themes consisted of both barriers and enablers, and have been labelled as relating to information delivery, or resource availability. The domain of *memory, attention, and decision processes* contained themes relating to both of these labels. Concepts within each theme remain domain-specific in spite of this considerable overlap, and were coded as either an enabler or barrier depending on participants’ descriptions.

Six theoretical domains that encapsulated the barriers and enablers to implementing national guidance for self-harm by general practice nurses: *knowledge* (*n* = 23 occurrences; reported by 10 [83%] participants); *cognitive and interpersonal skills* (*n* = 21 occurrences; reported by 9 [75%] participants); *memory, attention and decision processes* (*n* = 41 occurrences; reported by 11 [92%] participants); *environmental context and resources* (*n* = 26 occurrences; reported by 9 [75%] participants); *social influences*; (*n* = 28 occurrences; reported by 11 [92%] participants); and *professional role and identity* (*n* = 34 occurrences; reported by 12 [100%] participants). Participants described drivers relating to 5–11 domains each; those who reported the fewest domains had the shortest interview lengths. Additionally, all participants described at least three of the six salient theoretical domains, indicating that there were no deviant cases in the sample. Explanatory quotes are accompanied by anonymous participant ID in parentheses.

### Knowledge

#### Lack of access to CPD education (barrier)

Participants had undertaken no training about self-harm before qualifying for their current roles, and were not “*asked to evidence*” any education about mental health because “*it’s just assumed that [they] already know how to talk to people*” (Nurse Practitioner 9). Nurses did not receive education containing key information, such as “*looking out for the signs of [self-harm]”* (Practice Nurse 1). Participants perceived that such knowledge was essential for safeguarding: “*it comes down to whether or not you feel like you’ve got the knowledge to help that person to prevent them from self-harming*” (Practice Nurse 10). Difficulties accessing self-harm education included prohibitive cost barriers for practices, and insufficient continuous professional development (CPD) hours.

It isn’t something, and I’ve been nursing for 32 years, that I’ve ever received any training on. In my day, we did six-week psychiatric placements… that is it as far as my mental health education goes. (Practice Nurse 7).

#### Training needs (enabler)

Knowledge gaps were identified about warning signs for self-harm risk, and protocols following a disclosure or discovery of self-harm. A lack of knowledge about referral pathways, particularly “*where to refer to… and how to contact them*” (Practice Nurse 2), presented barriers to implementation. However, participants cautioned against “*in-depth learning experience[s] about self-harm*”, because “*there’s only so much that [they] can learn and do*” (Practice Nurse 7) as generalists; three participants described only requiring an “*awareness*” (Practice Nurse 5) of the self-harm guidelines. Comparisons were drawn with existing Education interventions, such as for manual handling; four participants suggested that information about self-harm could be delivered as part of annual safeguarding training “*because everybody has safeguarding training*” (Practice Nurse 10).

I’d expect, sort of, an awareness of signs and symptoms… to know the referral pathway, how soon that patient is to be seen… just the knowledge of who to go for what; which contact for what. (Practice Nurse 2).

### Cognitive and interpersonal skills

#### Building trust (enabler)

Participants were skilled at communicating with “*compassion, respect, and dignity*” (Practice Nurse 10). Rapport was deemed essential to “*broach [the] subject*” (Practice Nurse 12) of self-harm, but fostering long-term “*trust more than rapport*” (Practice Nurse 6) was considered important for continuity of care. There were opposing viewpoints about conversational techniques; five participants believed that patients would be reticent to “*admit*” (Practice Nurse 12) self-harm if asked directly, fearing that it could be interpreted as offensive. However, five other participants rejected strategies that they perceived to use hedging language, finding that patients are “*forthcoming if you’re just upfront and honest*” when asking about self-harm (Nurse Practitioner 9).

You also need to try and build that bond with the patients so that they’re engaging with you really, so that you can follow their care on… I think you also develop a skill of being able to listen to what they’re not saying as well. (Practice Nurse 6).

#### Challenging conversations (enabler)

Communication skills were considered to be products of experiential learning more than training, however, participants conceded that they sometimes experienced difficulties knowing what to say ‘on the spot’ to patients that spontaneously disclose self-harm. They identified a need for practical advice about appropriate things to say, and examples of “*open questions to ask*” to aid information-gathering (Practice Nurse 2). Potential solutions involved “*communication tools on how to actually start that conversation*” (Practice Nurse 1) about self-harm, or Training interventions that demonstrate how to communicate “*without patronising or judging*” (Practice Nurse 3) (i.e.: BCTTv1 6.1: Demonstration of the behaviour).

The sort of training that’s useful is the type [where] they tell you how to carry on the conversation with a person who’s starting to tell you about self-harm and how to react, and how to not back off it. (Practice Nurse 4).

### Memory, attention and decision processes

#### Translation of guidelines to support decision-making (enabler)

The guidelines were perceived to be useful to support decision-making about risk and referrals for self-harm, however, participants wanted the guidelines to be translated into more detailed protocols at a Trust- or service-level to ensure that “*everybody in the same service is doing the same thing*” (Practice Nurse 4). Barriers to decision-making included uncertainty about where to refer to, and the criteria for a referral. Other protocol-driven measures such as safeguarding processes, management plan pathways, and knowledge packs (e.g.: for falls) were identified as exemplar decision aids (i.e.: BCTTv1 12.5: Adding objects to the environment).

You need to have something specific for your area, that’s the thing, and that’s what NICE guidelines don’t do really. It’s just a very general overview. (Practice Nurse 5).

#### Recalling the guidelines and competencies (barrier)

Nurses described their assessment processes as “*engrained… right from the beginning of day one of your nurse training and the NMC guidelines*” (Practice Nurse 10). Although the self-harm guidelines were considered important, participants perceived that remembering their content was unfeasible due competing priorities in their workload, particularly relating to paid “*target*” incentives in primary care (Practice Nurse 7). Self-harm and mental health problems in general were framed as just one of several competing priorities encountered in general practice. Participants working in practices where self-harm encounters were infrequent expressed concerns that their “*knowledge of these guidelines [would] fade away*” without regular use (Practice Nurse 10). Interventions to combat forgetting were suggested, such as annual reminders accompanying safeguarding training, and intranet summaries of NICE guidance for quick-reference (i.e.: prompts and cues, BCTTv1 7.1).

We all strive to implement all our NICE guidelines, but there are so many of them… if I was constantly updating myself with NICE guidelines, that’s all I would be doing. I wouldn’t be seeing anybody. (Practice Nurse 7).

### Environmental context and resources

#### Time concerns (barrier)

Temporal barriers to guideline implementation were commonplace. Practice nurses reported difficulties with self-harm disclosures during appointments for other health concerns “*as they’ve got their hand on the door as they’re leaving*” (Practice Nurse 4), resulting in extended appointments that put pressure on colleagues to rearrange their patients, or cause other patients to be delayed. Further, there were concerns about referral waitlists; uncertainty about how long referrals take for non-urgent cases contributed to repeat appointments in primary care to monitor patients long-term.

You often find people are coming back, people’ll come back to me two, three, four times and say ‘I know I’m on the waiting list but it’s another two months or whatever before I’m seen.’ Whereas if you get them seen quicker, that would cut down our time. (Practice Nurse 4).

#### Supporting resources (barrier)

Communication with secondary mental health services was hampered by time-consuming paperwork: “*you write an essay about why you’re referring the patient*” (Practice Nurse 4). Although participants had access to same-day appointments from Crisis Teams for “*severe situations*” (Practice Nurse 10), concerns were raised about the availability of such services out of office hours, particularly at evenings and weekends. Nurses experienced challenges with information-sharing between services, particularly when following-up after referrals to confirm whether the patient contacted the service, or attended their appointment.

It’s very difficult nowadays for people to get appointments, even urgent ones… You would do what you could to get them an appointment that day. If necessary, I’d call the GP into the room so at least something was being done there and then. (Practice Nurse 5).

### Social influences

#### Supportive staff in general practices (enabler)

GPs supported practice nurses by providing team-based second opinions about patients. Two participants had a designated mental health or safeguarding lead they could consult about self-harm, while one participant identified a need for one in their practice. Support was enabled by an “*on-call system… [with] open door access*” (Nurse Practitioner 9) to a GP or another nurse, provided the practice had sufficient staff. Colleagues were enablers for implementation, through in-practice training, informal discussions, and structured all-staff meetings to highlight guideline updates and patients of concern: “*I think our practice is very good at communicating… it’s not just about GPs and then nurses separately. It’s a group thing.*” (Practice Nurse 10) (BCTTv1 3.2: Social support [practical]). One participant described having onsite access to a community-based psychiatrist part-time, which anecdotally improved communication with local community mental health services when requesting referrals.

One of the doctors I work with… I know he follows the guidelines which then makes it very easy to find out where the patient is on the pathway, so you know where to pick up from… That’s quite useful to have somebody who we regard as the fount of all knowledge. (Practice Nurse 4).

### Professional/social role and identity

#### Responsibilities and perceptions (enabler)

Participants were motivated to implement the guidelines because they felt a “*duty of care for [patients’] safety*” (Practice Nurse 6). Although some practice nurses did not have the responsibility to make mental health-related referrals in their practices, they acknowledged they still have an important role in signposting patients and raising concerns with GPs following encounters involving self-harm. Participants described patient perceptions that their profession made them more approachable to discuss sensitive topics like self-harm, due to perceptions that they are less busy than doctors. This enabled them to identify patients at risk of self-harm that could potentially be missed by their GP.

It’s just the perception of the nurse role. They’re there to care for patients, and that’s what they expect from us. They feel safe to share with us, and the perception that we listen better, whether that’s true or not I don’t know, but it’s what a lot of the patients still tell me. (Nurse Practitioner 9).

#### Uncertainty about professional role (barrier)

Nurses believed that their responsibilities towards patients had to be balanced by the limits of their expertise and whether or not it was “*appropriate*” to act (Practice Nurse 3). Knowledge gaps (described above) undermined participants’ professional confidence to implement the guidance, and they perceived that the expectations for nurses in general practice were unclear in the self-harm guidelines. Inability to conduct referrals to mental health services within practices was believed to arise from perceptions that practice nurses would not encounter patients who self-harm: “*they assume a patient that’s likely to self-harm or is self-harming would go straight to a GP. They don’t necessarily think that it’s going to be discovered incidentally… They don’t think to have a process in place because they assume it will go to them.”* (Practice Nurse 1). Seven participants described difficulties maintaining professional boundaries with patients they encountered that had self-harmed due to feelings of worry; one of these participants had a personal history of self-harm.

I think you need to come across caring but not too, you know, still within a professional boundary, to that effect. I think communication and the ability to ask in a sympathetic way really, that’s not something that’s everyone's got (Practice Nurse 1).

### Intervention development: proposed functions and exemplar behaviour change techniques

Descriptions of domains and exemplar quotes are presented in Table 2; candidate interventions were derived from relevant intervention functions and BCTs, which had been mapped according to the Behaviour Change Wheel. Five out of nine intervention functions [37] were linked to six TDF domains: education, training, environmental restructuring, enablement, and modelling. Nine of sixteen BCT groupings were found to be relevant: associations, natural consequences, comparison of behaviour, shaping knowledge, repetition and substitution, feedback and monitoring, antecedents, goals and planning, and social support. Sixteen unique BCTs were found to be relevant. For example, to target *memory, attention, and decision processes* suggested interventions might comprise: prompting nurses to rehearse and practice the process of making referrals for self-harm (intervention function: training; BCTTv1 8.3: Habit formation), or by adding an aide-memoire based on the self-harm guidelines to a practice’s intranet safeguarding guidelines for quick reference (intervention function: environmental restructuring; BCTTv1 12.5: Adding objects to the environment).

## Discussion

### Main findings

**Fig. 2:**
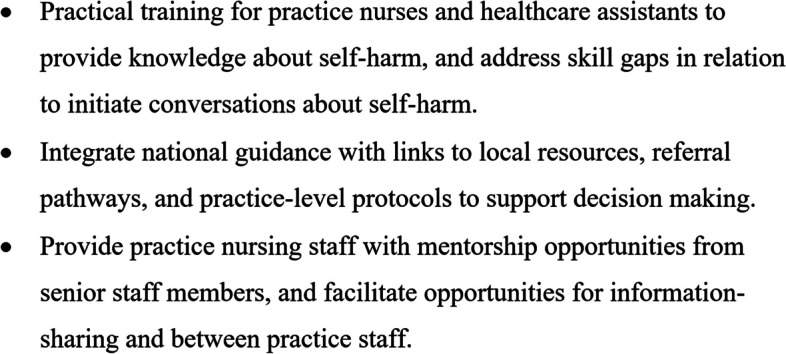
Summary of implications for practice, implementation and policy

This study contributes to the literature firstly by addressing the dearth of literature about primary care nurses’ experiences of suicide and self-harm prevention, and secondly by highlighting six distinct domains that summarise the challenges they face to implementing national guidelines with patients at risk of self-harm. Two key intervention targets were identified from converging explanatory themes in the data: information delivery, and resource availability. This is the first study to use a theoretically grounded framework to identify drivers of implementation of the NICE guidelines for self-harm among nursing staff working in primary care. The TDF framework [39, 40] was used to identify potentially modifiable barriers and enablers to inform the development of interventions to support guideline- and competency-adherent practice in primary care. We utilised the Behaviour Change Wheel [37] to provide recommendations for potential behaviour change techniques and intervention functions that could be incorporated into interventions to redress these targets, and support nursing staff in primary care to follow the NICE guidance for self-harm (summarised in Fig. 2).

Our findings complement previous research suggesting that few practice nurses have undertaken professional development about mental health topics, including self-harm, since qualifying [52, 53] (*Knowledge*). Although practice nurses perceive themselves to have an important role in activities related to mental health in primary care [52, 53] (*Professional Role and Identity*) they find communicating about emotionally charged topics to be challenging [54, 55], and believe that they need further training [56, 57] (*Cognitive and Interpersonal Skills*). Existing interventions to improve practice nurses’ communication skills have largely involved integrating motivational interviewing techniques into consultations [58]. While practice nurses utilise some of these techniques in intervention trials, they are not perceived to be easily applicable in routine practice [59, 60], suggesting a need for enablement in addition to skills provision. Although enhanced training and education is an opportunity for intervention, to support long-term recall and implementation of the national guidelines annual reminders or additional training may be needed to sustain behaviour [61] (*Memory, Attention, and Decision Processes*).

Consistent with our findings, practice nurses perceive a need for supporting resources, and encounter environmental barriers such as time constraints, workload, and cost barriers to accessing training [56, 62] (*Environmental Context and Resources*). As such, strategies are needed to create opportunities for practice nurses to be able to engage in professional development [52]. Uncertainty about referral processes and guideline content in other areas of clinical practice prevents practice nurses from implementing evidence-based practice [63, 64] (*Memory, Attention, and Decision Processes*). Lack of access to, and understanding of, primary information sources (such as national guidelines and research reports) reduces practice nurses’ capabilities to translate their acquired knowledge [65]. Environmental restructuring and enablement interventions could be designed to counter such uncertainty, to optimise the utility of the NICE guidelines in clear protocols that support decision-making without reinforcing template-driven care [63, 64]. An important resource described in our research was also a prominent enabler elsewhere in the literature; discussions and collaboration with colleagues was considered to be a powerful, and preferred, form of CPD among practice nurses [65–67] (*Social Influences*). Our findings suggest that routine support and information-sharing from other practice staff is a powerful enabler, which could be facilitated through modelling and enablement interventions [68]. However, intervention designers should be wary of pre-existing practice staff hierarchies, that potentially may be reliant on delegation and subordination instead of true collaboration [66, 69].

We also found that participants recounted differing experiences of encountering patient self-harm and judging risk based on the demographics of the patients; in this study, prominent examples included postpartum patients, and patients who were adolescents. Further research is needed to explore how differing patient characteristics may affect the way self-harm is responded to by primary care staff, such as patient age [70, 71], patients with young families [72], and patients with existing comorbidities [73, 74].

### Implications

The present study has shown the important role that nurses can have in recognising and responding to self-harm in primary care, and has identified candidate intervention potential approaches that would require further testing to support primary care staff to implement national guidelines for self-harm (summarised in Fig. 2). Firstly, staff would benefit from interventions to address deficits in knowledge and skills to support decision-making; sufficient education and training is needed to inform them about the content of the guidelines, self-harm risk factors, and to equip them with enhanced skills to tackle challenging conversations about mental health. Such interventions must be designed with generalists in mind; not only to prevent information overload and disengagement, but to ensure cost-effective and timely delivery within the scope of limited CPD hours [62, 67]. A potential solution may be to integrate self-harm into existing annual refresher training, such as for safeguarding practices, guided by existing competency frameworks [26, 75]. Within the context of the new self-harm guidelines [22], primary care nurses may need sufficient knowledge and skill to assess distress, intent, and the physical consequences of self-harm to make decisions about priority referrals. Secondly, resources are required to support adherence to the guidelines, that translate the guidelines into actionable decisions for practice nurses [65], and bolster collaboration and knowledge-sharing within general practices to ensure a uniform approach to self-harm [66, 69]. Resources that facilitate team-based continuity of care will be essential to implement the updated self-harm guidance; particularly to enable GPs and primary care nurses to coordinate regular reviews, follow-up appointments, and management of coexisting mental health problems [22].

This paper identified TDF domains from participant responses, to derive recommendations about specific BCTs that could form future interventions (represented in Table 2). Exemplar interventions could include peer coaching interventions to facilitate information-sharing about risk assessments and referral strategies (Intervention function: Modelling; BCT: Social support, practical). Interventions based on these findings would require piloting with involvement from practice nurses to provide acceptable, achievable improvements to guideline implementation. However, involvement with GPs, and key stakeholders such as practice managers, may also be fruitful to ensure interventions are feasible within general practices.

### Strengths and limitations

The present study had a number of limitations. Firstly, the sample was over-represented by older, more senior members of staff who are unlikely to have experienced recent training initiatives about self-harm and mental health. Future research should aim to investigate implementation among nurses who qualified after the addition of suicide prevention competencies, and compare their practice with more senior counterparts to evaluate whether the new curriculum facilitates best practice. The sample was also limited by a lack of men, who may encounter unique barriers or enablers to implementing the self-harm guidelines and nursing competencies [76]. The analysis did not involve any individuals with lived experience of self-harm or practice nurses; inclusion of such experts may have provided further insight into the salient drivers of guideline implementation by practice nurses. Discussion about certain TDF constructs, such as Emotion, were surprisingly absent from our data; although these domains may simply not be important influences on guideline implementation, since the topic guide was structured around the COM-B model instead of the TDF it is possible that some TDF constructs were overlooked by the wording of the COM-B-derived questions. Alternative analytical approaches such as grounded theory may have better identified emergent themes that are not sufficiently explained by the TDF. However, a strength of this research is that our data still demonstrates spontaneous emergence of TDF-aligned themes, which supports the validity of mapping TDF constructs to the Behaviour Change Wheel [39, 40]. By using a broad, semi-structured interview guide, participants had the opportunity to naturally describe drivers [45]. By utilising the TDF and BCT Taxonomy V1 together [77] we provide a theoretically-informed foundation for the development of quality improvement interventions, through a comprehensive collection of recommendations.

## Conclusions

Nursing staff in general practice are well-placed to recognise and respond to self-harm [78], but require support to adhere to national guidelines and nursing competencies. The present study used the Behaviour Change Wheel to [1] identify the drivers that influence whether practice nurses can implement the NICE guidelines for self-harm, and [2] suggest candidate interventions to support implementation, as derived from relevant TDF domains and behaviour change techniques. The six domains derived from the data could be addressed separately through targeted interventions, or together as part of more ambitious, complex interventions to improve quality. This work represents a starting point in addressing the lack of research around the roles of nursing staff in primary care for self-harm, and provide timely recommendations to support them to assess and manage patients at risk of self-harm.

### Supplementary Information

**Additional file 1.** Interview Schedule.

**Additional file 2.** Information for the Interviewer Guide.

## Data Availability

The dataset used and analysed during the current study are available from the corresponding author on reasonable request.

## Abbreviations

BCT: Behaviour Change Technique
BCTTv1: Behaviour Change Technique Taxonomy Version 1
COM-B: Capability, Opportunity, Motivation model of Behaviour
CPD: Continuous Professional Development
HM: Her Majesty’s (Government)
GDPR: General Data Protection Regulation
GP: General Practitioner
ID: Identity
NICE: National Institute for Health and Care Excellence
NMC: Nursing and Midwifery Council
TDF: Theoretical Domains Framework
UK: United Kingdom
