# Supplementary material for: Implementation of national guidance for self-harm among general practice nurses: a qualitative exploration using the capabilities, opportunities, and motivations model of behaviour change (COM-B) and the theoretical domains framework
Source: BMC Nurs. 2023 Dec 1;22:452. doi: 10.1186/s12912-023-01360-3 (PMC10693142; doi:10.1186/s12912-023-01360-3)
Supplement: Supplementary file 1 — Additional file 1. Interview Schedule. [file 12912_2023_1360_MOESM1_ESM.docx]

###### Additional file 1: Interview Schedule

**Interview Topic Guide developed by University of Manchester for YouGov**

**May / June 2019**

**Interview Topic Guide (30 – 45mins)**

**Intro text – standard YG text (3 mins)**

- Hello and thank you for taking part in this interview today, which is being conducted by YouGov on behalf the University of Manchester.
- Remind them that the interview will be made anonymous and we follow the GDPR. Explain that their responses would only be reported via their job role and not their name
- Depths will be audio recorded for note-taking purposes. Are you happy for us to share the recording with the research team at Manchester University (Psychology Dept)?
- There are no right or wrong answers, please try to be as open and honest as possible. You may refuse to answer any question at any time.

**Please start by introducing yourself – 1^st^ name and role / main responsibilities.**

- **How long have you been in your current role?**
- **How long have you been working in primary care in total?**

| **Question** |
| --- |
| **Your role and experience of self-harm (15 mins)** |
| 1. What role do you think a *<health professional in primary care>* plays in assessing or managing patients who are at risk of self-harm?  - Have you ever encountered a patient you thought was at risk of self-harm? - How often do you typically encounter patients who are at risk of self-harm? |
| 1. Do you see self-harm prevention as something you are personally responsible for? Why or why not?  - To what extent is it a priority for you? |
| 1. Do you have any **protocols** around the assessment and management of self in your place of work?  - **If not,** what are your views on this? Why do you think you do not have any formal protocols? - **If so,** during your time as a *<health professional in primary care>*, how has the **assessment and management** of self-harm in your practice/place of work changed if at all? - Have **protocols** changed over time? If so, how? |
| 1. Can you tell me about any **training** you have received for assessing or managing self-harm? E.g. type of training, provider and method e.g. f2f or online  - Did you find it useful for encountering patients at risk of self-harm? Please explain your reasons - Is there anything you would change about your training?   ***If no training***: What would you expect from training to prepare you for encounters with patients at risk of self-harm? |
| 1. Are you aware of any **tools** you might use when encountering a patient who is at risk of self-harm?  - What is the tool? How would it help you? - Could it be improved? If so, how?   *If no tools used*: Is there anything you can think of that would help you to assess and manage a patient at risk of self-harm? |
| 1. Please talk me through what you would typically do if you encountered a patient who you thought was ***at risk*** of self-harm.  - If relevant, please describe a past encounter (we understand that this needs to be kept anonymous) |
| 1. What do you think you would do if you encountered a patient who had ***actually*** self-harmed?  - If relevant, you can describe a past encounter (we understand that this needs to be kept anonymous) |
| 1. Please can you tell me about what you would do if you thought you needed to **refer** a patient who was ***at risk*** of self-harm, either for a psychosocial ***assessment*** or for a psychosocial ***intervention***? (*e.g. cognitive behavioural therapy*)  - Would you expect to encounter any difficulties with the process? If so, what type of difficulties? |
| **Role responsibility for implementing NICE guidelines for self-harm (7-10 mins)** |
| 1. Before you took part in this research were you **aware of the NICE guidelines** **for self-harm in primary care**? If so, how? E.g. at University, training, current workplace etc |
| 1. If you were aware of the guidelines: Would you say that generally you are in a habit of implementing the NICE guidelines for self-harm? Why or why not?  - What would be helpful in developing a routine/habit of implementing the NICE guidelines? |
| 1. What **skills** do you think are required to implement the NICE guidelines for self-harm?  - To what extent do you have these skills? - How did you acquire these skills (training or experience)? (OR: If they don’t feel they are skilled: What would help you to acquire the necessary skills?) |
| 1. Do you receive any **support from your colleagues** to implement the NICE guidelines for self-harm? If so, what role do the colleagues have?  - Are the guidelines promoted in your workplace? If so, how? - **If not**, what type of support would you like / need? |
| 1. To what extent does your work environment provide the opportunity to implement the NICE guidelines for self-harm?  - What would need to change about your workplace for you to better implement the guidelines? |
| **General perceptions of the NICE guidelines for assessing and managing self-harm (7 – 10 mins)** |
| 1. What do you think about the NICE guidelines for assessing and managing self-harm ***in general***?  - Any surprises? - Do you think they are suitable? Why/why not? |
| 1. What do you think about i**mplementing** the NICE guidelines for self-harm ***in your role/place of work***? Do you think they are suitable? Why/why not?  - How easy is it for you to implement the NICE guidelines in your workplace? |
| 1. What are / do you think would be the main **challenges** of implementing the NICE guidelines for self-harm with patients?  - Is there anything specific that prevents you from implementing the guidelines? |
| 1. What do you think are the **benefits** of implementing the NICE guidelines for assessing and managing self-harm?  - *To patients* - *To you personally* |
| **Suggestions (3 mins)** |
| 1. Is there anything that we haven’t covered that you feel is important/relevant? |
