# Supplementary material for: Implementation of national guidance for self-harm among general practice nurses: a qualitative exploration using the capabilities, opportunities, and motivations model of behaviour change (COM-B) and the theoretical domains framework
Source: BMC Nurs. 2023 Dec 1;22:452. doi: 10.1186/s12912-023-01360-3 (PMC10693142; doi:10.1186/s12912-023-01360-3)
Supplement: Supplementary file 2 — Additional file 2. Information for the Interviewer Guide. [file 12912_2023_1360_MOESM2_ESM.docx]

###### Additional file 2: Information for the Interviewer Guide

**Interview Topic Guide developed by University of Manchester for YouGov**

**May / June 2019**

**Information for the Interviewer to read before the interview**

**Definitions:**

- **Self-harm**: Intentional self-poisoning or self-injury, ***irrespective of motive*** or the ***extent of suicidal intent***. Self-harm is an expression of personal distress, not an illness, and there are many varied reasons for a person to harm him or herself.
- **Patient *at risk* of self-harm**: Any patient who you think ***might harm themselves***, including patients who have a ***prior history*** of self-harm, and patients who have ***never self-harmed before***.
- **Protocols**: ***In addition*** to the national guidelines, individual practices may have developed their ***own protocols*** to follow when a patient presents with self-harm.
- **Tools**: Any ***resource*** that healthcare professionals can utilise when encountering a patient at risk of self-harm. This includes any ***risk assessment forms*, *care pathways/flowcharts*, or a *brief intervention***.
- **Refer a patient at risk of self-harm**: If there is not a **s*uitably qualified/trained*** healthcare professional ***onsite*** to conduct a comprehensive psychosocial assessment, the patient must be referred to a mental health specialist for assessment. Following psychosocial assessment, a patient may need to be ***referred for further treatment*** (such as a psychosocial intervention).
- **Psychosocial assessment**: A comprehensive assessment including an ***evaluation of* *needs and risk***. The assessment of needs is designed to ***identify psychological and environmental (social) factors*** that ***might explain an act of self-harm***.
- **Psychosocial intervention**: An ***activity or therapy*** designed to change specific thoughts and behaviours, such as cognitive behavioural therapy (CBT) or dialectal behavioural therapy (DBT).
- **NICE**: The National Institute for Health and Care Excellence (NICE) is a non-departmental public body that ***provides national guidance and advice*** to improve health and social care in England.
- **NICE Guidelines**: ***Evidence-based recommendations*** on a wide range of topics, from preventing and managing specific conditions, improving health, and managing medicines in different settings, to providing social care and support. They aim to promote ***individualised care and integrated care***.

**Outline of the guidelines for primary care:**

**NICE Guidelines for self-harm**: The NICE guidelines for self-harm are split into short-term and long-term management for all patients aged 8 years old and older. This guide refers to ***short-term management*** of self-harm, specifically for professionals working in ***primary care***. The guidelines recommend that any healthcare professionals who ***may*** ***have to assess and/or treat*** people who have self-harmed should ensure that they are ***properly trained*** and ***competent to undertake assessment and treatment as necessary.***

1. When a patient presents in primary care following an episode of self-harm, healthcare professionals should ***establish the likely physical risk***, and the person's ***emotional and mental state***, in an atmosphere of respect and understanding. Patients should be referred for urgent treatment in an emergency department if assessment suggests there is a significant risk.
2. Risk assessment should include ***identification of the main clinical and demographic features*** and ***psychological*** ***characteristics known to be associated with risk***, in particular depression, hopelessness and continuing suicidal intent.
3. If urgent referral to the emergency department is not necessary, a ***risk and needs*** (psychosocial) assessment should be undertaken to assess the case for urgent ***referral to secondary mental health services***. This should be comprehensive and include evaluation of the ***social, psychological and*** ***motivational factors specific to the act of self-harm***, current intent and hopelessness, as well as a full mental health and social needs assessment.

Following assessment and treatment of self-harm in primary care, the outcome of the risk and needs assessment, and full details of the treatment provided, should be ***forwarded to the appropriate secondary mental health team*** at the earliest opportunity. Patients and the assessor should read through the assessment where possible, to ***agree the assessment and care plan***.
